# Evaluating the effectiveness of a clinical practice change intervention in increasing clinician provision of preventive care in a network of community-based mental health services: a study protocol of a non-randomized, multiple baseline trial

**DOI:** 10.1186/1748-5908-8-85

**Published:** 2013-08-06

**Authors:** Kate Bartlem, Jennifer Bowman, Megan Freund, Paula Wye, Kathleen McElwaine, Jenny Knight, Patrick McElduff, Karen Gillham, John Wiggers

**Affiliations:** 1 Population Health, Hunter New England Local Health District, Booth Building, Wallsend Health Services, Longworth Avenue, Wallsend, NSW 2287, Australia; 2 School of Medicine and Public Health, Faculty of Health, The University of Newcastle, University Drive, Callaghan, NSW 2308, Australia; 3 Hunter Medical Research Institute, Clinical Research Centre, Level 3 John Hunter Hospital, Lookout Road, New Lambton Heights, NSW 2305, Australia; 4 School of Psychology, Faculty of Science and Information Technology, The University of Newcastle, University Drive, Callaghan, NSW 2308, Australia

**Keywords:** Practice change, Clinical practice change, Mental health, Mental health services, Community mental health, Preventive care, Smoking, Nutrition, Alcohol, Physical activity

## Abstract

**Background:**

People with a mental illness experience substantial disparities in health, including increased rates of morbidity and mortality caused by potentially preventable chronic diseases. One contributing factor to such disparity is a higher prevalence of modifiable health risk behaviors, such as smoking, inadequate fruit and vegetable intake, harmful alcohol consumption, and inadequate physical activity. Evidence supports the effectiveness of preventive care in reducing such risks, and guidelines recommend that preventive care addressing such risks be incorporated into routine clinical care. Although community-based mental health services represent an important potential setting for ensuring that people with a mental illness receive such care, research suggests its delivery is currently sub-optimal. A study will be undertaken to evaluate the effectiveness of a clinical practice change intervention in increasing the routine provision of preventive care by clinicians in community mental health settings.

**Methods/design:**

A two-group multiple baseline design will be utilized to assess the effectiveness of a multi-strategic intervention implemented over 12 months in increasing clinician provision of preventive care. The intervention will be implemented sequentially across the two groups of community mental health services to increase provision of client assessment, brief advice, and referral for four health risk behaviors (smoking, inadequate fruit and vegetable consumption, harmful alcohol consumption, and inadequate physical activity). Outcome measures of interest will be collected via repeated cross-sectional computer-assisted telephone interviews undertaken on a weekly basis for 36 months with community mental health clients.

**Discussion:**

This study is the first to assess the effectiveness of a multi-strategic clinical practice change intervention in increasing routine clinician provision of preventive care for chronic disease behavioral risk factors within a network of community mental health services. The results will inform future policy and practice regarding the ability of clinicians within mental health settings to improve preventive care provision as a result of such interventions.

**Trial registration:**

Australian and New Zealand Clinical Trials Registry (ANZCTR) ACTRN12613000693729.

## Background

Modifiable health risk behaviors are the largest contributor toward the burden of chronic disease morbidity and mortality in Australia and internationally [1,2]. In particular, smoking, inadequate nutrition, harmful alcohol consumption, and inadequate physical activity, are the primary behavioral risks for the most common causes of preventable morbidity and mortality [3,4]. While the prevalence of such health risk behaviors is reported to be high among the general population [2,5], the prevalence is generally higher still among people with a mental illness [6-8].

The provision of preventive care by health services, including mental health services, has been suggested as an important opportunity to address the burden of chronic disease [9-12]. Such preventive care has been recommended to follow the 5A’s model [13]; however, given time constraints and competing clinical priorities, an abbreviated model of ‘2As and an R’ (assessment, brief advice, and referral) has been suggested as appropriate [14-16]. Further, the value of addressing multiple health risk behaviors concurrently has been suggested to be a cost-effective means of both implementing and adopting behavior change strategies [13,17].

Though limited research has examined the provision of such care within mental health services, that which has been conducted suggests that its delivery is sub-optimal in both Australia [18-20] and elsewhere [21-27]. It is increasingly recognized that for clinician provision of preventive care to be enhanced, practice change interventions must address the clinical practice systems and procedures within which clinicians work [28]. Within health settings generally, it is suggested a number of clinical practice change strategies are effective in improving the quality of healthcare and clinician adherence to guidelines, including: local opinion leaders [29], audit and feedback [30], reminders and clinical decision support systems [31], printed education materials including clinical practice guidelines [32], and training and education [33]. Limited research has examined the effectiveness of such interventions in increasing the delivery of preventive care in mental health settings; and that which has been conducted has occurred within the inpatient setting only, has not aimed to increase the routine provision of preventive care by clinicians, or has not involved controlled research designs [34-36].

Community-based mental health services represent an appropriate setting for the provision of preventive care to clients in a number of countries [37,38]. In Australia, such services reported over 7.1 million client visits in 2010 to 2011, and provided care to a greater number of clients than did psychiatric inpatient settings [39]. A number of the characteristics of care delivery in this setting have the potential to enhance successful health behavior change, including: the capacity for preventive care to be provided over multiple visits due to the frequency of contact with clients, the existence of a multidisciplinary team of health professionals skilled in behavioral management, care delivery occurring in a variety of settings including a client’s home [40], and the opportunity to refer clients to evidence-based specialist preventive care services as required, including telephone helplines [41].

To the authors’ knowledge, no research has examined the effectiveness of a clinical practice change intervention in increasing preventive care provision within a community-based mental health service setting. To address this gap, a study is proposed to determine the effectiveness and acceptability of a multi-strategic clinical practice change intervention in increasing community mental health clinicians’ provision of preventive care (risk assessment, brief advice to modify behaviors, and referral to ongoing behavior change support services) to clients for four behavioral health risk factors (smoking, inadequate fruit and vegetable consumption, harmful alcohol consumption, and inadequate physical activity) across a network of community mental health services.

## Methods/design

### Study design and setting

**Figure 1:**
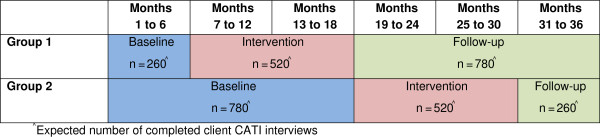
Overview of study design.

The study will be undertaken within community-based mental health services across one local health district in New South Wales, Australia. A two-group multiple baseline design [42] will be utilized to assess the effectiveness of an intervention implemented over 12 months (Figure 1). The two groups will be defined according to geographic and administrative service boundaries. The intervention will be implemented sequentially across the two groups, and involve clinical practice change strategies to increase clinician provision of three elements of care for clients (assessment, brief advice, referral) for four health risk behaviors (smoking, inadequate fruit and vegetable consumption, harmful alcohol consumption, and inadequate physical activity).

Primary data collection will consist of repeated cross-sectional computer-assisted telephone interviews (CATIs) undertaken with clients receiving care from the community mental health services. The interviews will measure client-reported receipt of preventive care on a weekly basis for 36 months. Surveys will commence in both groups 6 months prior to the intervention implementation in the first group of services, and continue until 6 months after the 12 months of intervention in the second group of services. To supplement the client data, CATIs will be undertaken with community mental health clinicians, pre and post the 12 months of intervention in each group, to measure clinician self-reported preventive care provision. Intervention effectiveness will be demonstrated by an increase in the prevalence of clients reporting the receipt of preventive care from the community mental health services following the 12 months of intervention in each group, and an increase in clinician-reported provision of preventive care to their clients, relative to the baseline period.

### Participants

#### Community mental health facilities

All community mental health facilities in the health district providing care to adult clients (19 services) will receive the clinical practice change intervention. Community mental health care services provided by such facilities include general adult community mental health services, and specialized community mental health services, including older persons services and psychiatric rehabilitation. Types of community mental health care services excluded from participating in the study will include inpatient and intake triage services, and services providing care solely to clients under the age of 18.

#### Clients

Clients eligible for participation in the CATI interviews will be those who: are 18 years or older; have attended at least one face-to-face individual appointment with a community mental health clinician within the previous two weeks; have not previously been selected to participate; and have not been identified by their clinician as inappropriate to contact. Additional client eligibility criteria will be assessed upon phone contact and will include: English speaking; not participating in any other surveys regarding health behaviors or care received at community health services; not living in aged care facilities or jail; and being physically and mentally capable of responding to the survey items.

#### Clinicians

All clinicians within eligible services will receive the intervention. This includes multidisciplinary teams of psychiatrists, psychologists, social workers, dieticians, nurses, and occupational therapists. All clinicians who have had a minimum of 10 individual face-to-face appointments with adult clients within the two months prior to the survey, have been employed for a minimum of three months, and are not contractors are eligible for participation in the CATI interviews.

### Recruitment

#### Clients

Each week, for 36 months, a random sample of 40 adult clients (20 from each of the two groups) who had received care at the eligible community mental health services in the prior two weeks, will be selected from electronic medical records and invited to participate in the data collection.

#### Clinicians

Clinician surveys will be undertaken pre and post the intervention delivery for each of the two groups. All eligible community mental health clinicians providing care to clients at the community mental health services (approx. n = 200) will be identified through electronic medical records and will be invited to participate in the data collection.

### Intervention model

#### Model of preventive care

As limited clinician time is cited as a barrier to providing preventive care in general primary care settings [13,43,44] and mental health care settings [45,46], it has been recommended that the 5A’s (ask, advise, assess, assist and arrange) model of preventive care be shortened to ‘2As and an R’, whereby clinicians ask, advise and refer clients on for specialized further care [13,44,47,48]. This approach emphasizes referral of clients to specialist services rather than clinicians providing the extended care themselves, thereby limiting the demand on clinicians’ time, and encouraging client access to specialized health behavior change interventions. Based on these recommendations, the study will focus on increasing the routine provision of preventive care, following the ‘2As and an R’ model [13,44,47,48].

#### Assessment

Clients will be screened for each of the four health risk behaviors based on risk levels defined in Australian national guidelines and recommendations. Clients will be defined as being at risk according to the following: any tobacco smoking [49], consuming less than two servings of fruit or five servings of vegetables per day [50], consuming more than two standard drinks on average per day, or four or more standard drinks on any one occasion [51], or engaging in less than 30 minutes of physical activity on at least five days of the week [52]. Inadequate fruit and vegetable consumption were selected as nutrition indicators due to their emphasis within the national guidelines, and associations with lower chronic disease morbidity and mortality, including evidence of a protective effect against cardiovascular diseases, diabetes, and some cancers [53-55].

#### Brief advice

Clients identified as being at risk for any of the health risk behaviors according to the above definitions of risk, will be provided brief advice regarding their identified health risk behavior; including advice on how to modify their risk to comply with the Australian national guidelines, and the benefits of doing so.

#### Referral

All clients identified as at risk for any of the health risk behaviors will be offered a referral to specialized support services. Where available, clients will be referred to the evidence-based, state-wide telephone support services New South Wales (NSW) Quitline for smoking, and the NSW Get Healthy Information and Coaching Service for inadequate fruit and vegetable intake and inadequate physical activity. No equivalent service is currently available for clients identified as at risk for harmful alcohol consumption. Clients identified as having risks will also be prompted to see their General Practitioner (GP) or Aboriginal Medical Service for further assessment, care and follow-up. Additional referrals (for example; local dietician, community exercise group, drug and alcohol service) may be made according to clinician judgement, or because the referral avenue may be more culturally appropriate for Aboriginal or Torres Strait Islander clients.

#### Clinical practice change intervention

The multi-strategic clinical practice change intervention will be implemented at each community mental health service to support the provision of preventive care delivery. The practice change intervention is informed by extensive practice change research and reviews of the clinical practice change literature [29,30,32,33,56] and will include the following:

##### 1. Leadership and consensus

District-wide policy guidelines and policy compliance procedures will be implemented to formalize the intervention and increase adherence. Existing clinical networks, clinical sites and teams will be engaged and consulted prior to and during the implementation of the intervention. Consultation will be undertaken with high level management regarding their advocacy, leadership and support of the intervention, and to gain agreement on Key Performance Indicators. At each clinical site, managers and clinicians will be consulted regularly throughout the intervention.

##### 2. Enabling systems and procedures

Modifications will be made to the existing medical record software routinely used by all community mental health clinicians. A standardized electronic assessment tool will be incorporated into the medical record to enable the standardized provision and recording of: risk assessment for each health risk behavior, brief advice on how to improve behaviors in order to meet the Australian national guidelines (where a client is at risk), referral to the recommended referral services and/or additional local referral avenues (where a client is at risk), automated production of a tailored client information handout regarding health risk behavior, advice, and referral, and automated production of a referral letter to clients’ GP or Aboriginal Medical Service regarding care provided during the appointment(s). The standardized tool will prompt assessment for each of the health risk behaviors, and based on the risk assessment information entered, will prompt advice and referral where a client has a risk.

##### 3. Clinician and manager training

Clinicians will be required to complete online educational competency-based training modules covering the importance of providing preventive care, information on the policy guidelines and Key Performance Indicators, the model of preventive care, and the recording of such care in the standardized electronic assessment tool. The online training modules will take approximately two hours to complete, and will be followed by a brief competency-based multiple choice quiz. Clinicians will be considered trained upon the completion of the quiz, with a score of 100% attained. Managers of each service will be required to attend a two-hour, face-to-face training session covering the importance of providing leadership in preventive care, and education around the performance monitoring and feedback strategy, including interpretation and use of preventive care performance reports.

##### 4. Monitoring and feedback

Modifications will be made to the existing electronic medical record software to allow automated production of preventive care performance reports. The reports will include the proportion of clients assessed, and of those identified at risk, the proportions provided brief advice and offered referral. Reports will be provided to and discussed with managers on a monthly basis. Report discussions will be linked to the district-wide Key Performance Indicators and will focus on developing strategies to improve performance where required. Existing district-wide quality assurance systems will also be modified to incorporate preventive care indicators.

##### 5. Provision of practice change resources

All clinicians and managers will receive a preventive care resource pack to assist with delivery of care. Resources within these packs include: a preventive care process flowchart, a guide for providing and recording care within the electronic medical record software, information on fruit and vegetable serving sizes and standard alcoholic drinks, fax-based referral forms for the Quitline and Get Healthy service, a preventive care flipchart to use as a visual aid during care provision, and paper-based preventive care assessment tools for the delivery and recording of preventive care when away from a computer. All clinicians will be provided with monthly newsletters and tip-sheets, and access to an e-mail helpline and internet resource site. Each service will be provided a clinical practice change support officer to support intervention delivery, and provide a minimum of fortnightly phone calls and/or e-mails to support managers in implementing and maintaining preventive care delivery, and monthly face-to-face visits to support managers and clinicians.

### Data collection procedures

#### Client CATI

Selected clients will be mailed an information statement informing them of the survey and data collection procedures, and providing them with a toll free number that they can call should they not wish to be contacted for participation. Trained interviewers, blind to group allocation, will contact the remaining selected clients by phone approximately two weeks later, and ask whether they would like to participate. Consenting clients will be administered the interview, or a more suitable time for conduction of the interview will be arranged.

#### Clinician CATI

Eligible staff will be mailed an information statement informing them of the survey and data collection procedures, and phoned by trained interviewers approximately four weeks later during work hours and asked to participate in the study. Consenting clinicians will be administered the interview, or a more suitable time for conduction of the interview will be arranged.

### Measures: client CATI

#### Client characteristics

Clients will be asked to report their: Aboriginality (Aboriginal, Torres Strait Islander, both, neither), highest education level attained (never attended school, some primary school, primary school, some high school, school certificate or equivalent, High School Certificate or equivalent, TAFE certificate or diploma, university degree or higher), current employment status (full time, part-time/casual, unemployed, can’t work for health reasons, home duties, student, retired, other), current marital status (never married, married or living together in a relationship, separated, divorced, widowed), and any physical or psychiatric conditions for which they have received care within the previous two months. Further demographics of age, gender, postcode, and number of community mental health appointments within the last 12 months will be attained from electronic medical records.

#### Client risk status

Clients will be asked to report on their health behavior risk status during the month prior to seeing their community mental health clinician. Clients will be asked:

1. If they smoked any tobacco products (yes–daily; yes–less than once a week; not at all–quit less than 4 months ago; not at all–quit 4 months or more ago; not at all-never smoked);

2. How many serves of fruit (0, 1, 2 or more) and vegetables (0, 1, 2, 3, 4, 5 or more) they usually ate per day;

3. How often they had a drink containing alcohol (never; monthly or less; 2 to 4 times a month; 2 to 3 times a week; 4 or more times a week); and for those who had consumed alcohol during the month prior to seeing their community mental health service, how many standard drinks they consumed on a typical drinking day (1 to 2; 3 to 4; 5 to 6; 7 to 9; 10 or more) and how often they consumed four or more standard drinks on one occasion (never, less than monthly, monthly, weekly, daily or almost daily); and

4. How many days a week they usually undertook 30 minutes or more of physical activity (0; 1; 2; 3; 4; 5 or more; can’t for health or treatment reasons).

In line with Australian National Guidelines, clients will be defined as being at risk if they report: smoking any tobacco products [49], consuming less than two servings of fruit or five servings of vegetables per day [50], consuming more than two standard drinks on average per day, or four or more standard drinks on any one occasion [51], or engaging in less than 30 minutes of physical activity on at least five days of the week [52].

#### Provision of preventive care

Assessment: Clients will be asked to report whether, during their community mental health appointments, the clinician asked about their smoking status, fruit and vegetable intake, alcohol consumption, and physical activity (yes, no, don’t know for each).

Brief Advice: Clients classified as at risk for a health risk behavior(s) will be asked whether their community mental health clinician advised them to modify their behavior(s) (yes, no, don’t know for each).

Referral: Clients classified as at risk for a health risk behavior(s) will be asked whether their community mental health clinician: (yes, no, don’t know for each)

1. Spoke to them about the NSW Quitline telephone support service (for clients at risk for smoking);

2. Spoke to them about the NSW Get Healthy Coaching and Information Service (for clients with inadequate fruit and vegetable intake or inadequate physical activity);

3. Offered to arrange for a telephone support service (NSW Quitline or NSW Get Healthy Coaching and Information Service) to call them; and if so, whether the client accepted this offer;

4. Recommended that they speak to their GP or Aboriginal Medical Service about their health risk behavior(s);

5. Advised them to use any other supports to make changes to their health behavior(s) (for example, a dietician, physical activity classes, support groups, or internet websites), and what support was advised; and

6. Offered to send their GP or Aboriginal Medical Service a letter summarizing their health behavior risks and the preventive care provided.

#### Acceptability of preventive care delivery

Clients will be asked to respond on a five-point Likert scale their agreement with statements regarding each element of care for each health risk behavior (strongly disagree to strongly agree). For example, ‘It is acceptable for the service to ask you about how much alcohol you drink.’ Clients will additionally be asked to respond on a five-point Likert scale their agreement with statements regarding receiving each element of care for all four health risk behaviors in the one appointment (strongly disagree to strongly agree).

### Measures: clinician CATI

#### Clinician characteristics

Clinicians will be asked to report their age (20 to 29, 30 to 39, 40 to 49, 50 to 59, 60 to 64, 65 to 69, 70+), Aboriginality (Aboriginal, Torres Strait Islander, both, neither), years in their current discipline (1 to 2, 3 to 4, 5 to 9, 10+), and their current employment status (full time, part time, casual, other). Clinicians will further be asked to report on their own health behavior risk status during the month prior to the interview. Additional clinician characteristics including service type and discipline type will be obtained through the electronic medical records system.

#### Preventive care delivery

On a scale of 0% to 100%, clinicians will be asked to estimate over the previous two months the proportion of new adult clients they provided with the following:

1. Assessment: the proportion for which they assessed their smoking status, fruit and vegetable intake, alcohol consumption, and physical activity;

2. Brief Advice: For clients with health risk behaviors, the proportion to which they provided brief advice to modify that behavior(s); and

3. Referral: For clients with health risk behavior(s), the proportion they spoke to about the telephone support services, the proportion they arranged for a telephone support service to call them, the proportion they recommended speak to their GP or Aboriginal Medical Service about that behavior(s), the proportion they advised to use any other supports to make changes to their health behavior(s), and the proportion they offered to send a summary of their health risks behavior(s) to their GP or Aboriginal Medical Service.

#### Attitudes and beliefs towards preventive care

Clinicians will be asked to respond on a five-point Likert scale their agreement with a number of statements related to their attitudes and beliefs towards providing preventive care to their clients (strongly disagree to strongly agree). Items will relate to: whether clients find preventive care acceptable, clinician confidence in providing preventive care, availability of services to refer clients to, whether clients will change their behaviors as a result of the care they provide, management support regarding the delivery of preventive care, knowledge and skills, role congruence, time available to provide preventive care, whether preventive care will jeopardize relationships with clients, and whether clients are interested in changing health behaviors.

#### Resources and supports available for preventive care delivery

Clinicians will be asked whether there are resources and supports available to assist in the delivery of preventive care (yes, no, don’t know), and where available, the usefulness of that resource or support (very useful, somewhat useful, not at all useful). Resources and supports will include printed educational materials (*e.g*., hard copy resources), educational meetings (*e.g*., training), local opinion leaders (*e.g*., a staff member to support preventive care), audit and feedback (*e.g*., feedback on preventive care provided within the service), and reminders or prompts (*e.g*., list of referral services).

#### Sample size and power

Pilot data indicate an expected completion rate of 50% for the primary data collection (client CATI). Based on this completion rate, the estimated sample size is as follows: 1,040 participants during the baseline periods (260 from group 1; 780 from group 2), 1,040 participants during the intervention periods (520 from each of group 1 and group 2), and 1,040 participants during the follow-up periods (780 from group 1; 260 from group 2) (see Figure 1).

Based on a 50% prevalence of care at baseline, and with before and after samples of approximately 1,040 clients, the study will have 80% power to detect a 7% increase in the provision of assessment of risk behaviors at the 1.67% significance level. An alpha level of 0.0167 has been chosen because there are three primary endpoints to be tested (assessment for all behaviors, advice for all behaviors, and referral for all behaviors). Analysis of changes in the provision of brief advice or offer of referral will be undertaken only for clients who report a risk factor. Based on the least prevalent risk factor (50% of clients at harm for harmful alcohol consumption), there will be before and after samples of approximately 520 clients at risk. With a presumed prevalence of care at baseline of 50%, the study will have 80% power to detect a 10% increase in the provision of brief advice and offer of a referral.

### Statistical analysis

Descriptive statistics will be used to describe client and clinician characteristics, risk status, prevalence of preventive care provided, client acceptability of preventive care, clinician attitudes and beliefs toward preventive care, and clinician-reported availability of resources and supports for preventive care delivery.

#### Client CATI

Logistic regression will be used to examine changes in preventive care delivery from baseline to follow-up, across the two groups combined. The models will examine change in each of the behaviors and change in all four behaviors combined. In the first set of models, the outcome of interest will be the client-reported assessment of risk behavior. The predictor variable of interest will be time (*i.e*., before/after) and we will include the covariates of age, gender, and number of visits to the service in the prior 12 months to account for any introduced selection bias [57] and group.

The second set of models will be restricted to those subjects who report risk behaviors, with the outcomes of interest being whether the community mental health clinician was reported to have provided brief advice or offer of a referral.

As simple random sampling of community mental health clients from a complete list of all clients who attended at least one face-to-face individual appointment with a community mental health clinician within the previous two weeks was used (see Recruitment), there is no need to adjust for clinician, community mental health service, or any other natural clustering that occurs within the community. An unadjusted analysis will provide an unbiased estimate of the statistics of interest.

#### Clinician CATI

Chi square tests will be used to compare preventive care provision, and attitudes and beliefs towards preventive care prior to and following the intervention.

### Ethics approval

Ethical approval to conduct the study has been obtained from the Hunter New England Human Research Ethics Committee (approval No. 09/06/17/4.03) and University of Newcastle Human Research Ethics Committee (approval No. H-2010-1116).

### Trial status

The intervention is currently underway within the second group of services.

## Discussion

To the authors’ knowledge, this is the first study to assess the effectiveness of a multi-strategic clinical practice change intervention in increasing clinician provision of preventive care for behavioral risk factors of chronic disease across a network of community mental health services. This study is an important step towards redressing the disparity in preventable health outcomes for those with mental illness, through the provision of a potentially sustainable practice change intervention aiming to improve clinical care for four behavioral health risk factors that contribute substantially to the increased rates of chronic disease morbidity and mortality experienced by this group. The results will inform future policy and practice regarding the ability of clinicians within mental health settings to improve preventive care provision as a result of such interventions.

## Abbreviations

CATI: Computer Assisted Telephone Interview; GP: General Practitioner; NSW: New South Wales.

## Competing interests

The authors declare that they have no competing interests.

## Authors’ contributions

Author KB led the manuscript development. Authors JK, MF, KG and JW conceived the intervention concept. All authors read and approved the final manuscript.
